# A case of post-traumatic acute appendicitis: uncommon consequence of blunt abdominal trauma

**DOI:** 10.1093/jscr/rjae761

**Published:** 2024-12-02

**Authors:** Norah I Alabdulaaly, Shahad Alghamdi, Mashael Albahli, Hisham Alabdullah, Abdullah Alghuliga

**Affiliations:** General Surgery Department, Prince Sultan Military Medical City, Ali ibn Saadah, Al Rayan, 4245, Riyadh 14212, Kingdom of Saudi Arabia; General Surgery Department, Prince Sultan Military Medical City, Ali ibn Saadah, Al Rayan, 4245, Riyadh 14212, Kingdom of Saudi Arabia; General Surgery Department, Prince Sultan Military Medical City, Ali ibn Saadah, Al Rayan, 4245, Riyadh 14212, Kingdom of Saudi Arabia; Trauma and Acute Care Surgery Department, Prince Sultan Military Medical City, Ahmed bin Alshaheed, Al Malaz, 3558, Riyadh 12832, Kingdom of Saudi Arabia; Trauma and Acute Care Surgery Department, Prince Sultan Military Medical City, Ahmed bin Alshaheed, Al Malaz, 3558, Riyadh 12832, Kingdom of Saudi Arabia

**Keywords:** trauma, appendicitis, abdominal blunt trauma

## Abstract

Acute appendicitis is known to be caused by intraluminal obstruction of the appendiceal lumen. Nonobstructive causes of acute appendicitis are rarely reported. Posttraumatic appendicitis is uncommon, and few cases have been reported in the literature. We present a case of acute appendicitis in a 21-year-old male confirmed via a computed tomography scan 5 days after a blunt abdominal injury. A laparoscopic appendectomy was performed, and the diagnosis of acute perforated appendicitis and peri appendicitis was confirmed histopathologically. The patient had an uneventful recovery following surgery.

## Introduction

Appendicitis is a common condition that requires urgent surgical intervention [1]. Inflammation of the appendix typically results from appendiceal lumen obstruction or nonobstructive processes that induce the migration of the intestinal flora to the appendicular wall, ultimately resulting in an inflammatory condition [2]. Delayed presentation and management of appendicitis may lead to complications, such as perforation, peritonitis, or intraabdominal abscess. Traumatic blunt abdominal trauma may lead to damage to the digestive tract or solid organs [3]. Few cases of traumatic appendicitis have been published; however, the pathophysiology of traumatic appendicitis is still debatable. Here, we present a case of acute appendicitis following blunt abdominal trauma in a male patient aged 21 years.

## Case report

A 21-year-old male presented to the emergency department with a history of right lower quadrant (RLQ) abdominal pain stabbing in nature, with sudden onset and progressive in severity. The patient experienced nausea, vomiting, and loss of appetite, and the pain was worsened by eating and was not alleviated by analgesics. The patient reported no history of fever, chills, weight loss, urinary issues, or diarrhea. His medical history revealed no significant chronic illnesses, and his surgical history was unremarkable. Additionally, no similar issues were reported in his family history. The patient had a history of blunt abdominal trauma after a road traffic accident 5 days before presentation, with a negative primary and secondary survey. The RLQ pain started after the trauma and did not improve. The patient was admitted to another hospital at the time of trauma, during which a pan-computed tomography (CT) scan was performed, and it was positive for grade 1 splenic injury and free pelvic fluid and negative for any other traumatic injuries. Therefore, based on the patient’s CT findings and medical report, he was admitted for 24 h of observation, and he was gradually started on a diet and was subsequently discharged in good condition. After discharge, the patient reported having on- and off RLQ abdominal pain, which was mild in severity at the beginning and then became more severe.

On examination, his vital signs were stable, and he was afebrile. The patient was generally in pain with no distress. His abdominal examination revealed no previous surgical scars. The abdomen was soft, with tenderness in the RLQ and positive rebound tenderness with no palpable mass. Per rectal (PR) examination has revealed good anal tone and no bleeding or palpable masses.

**Figure 1:**
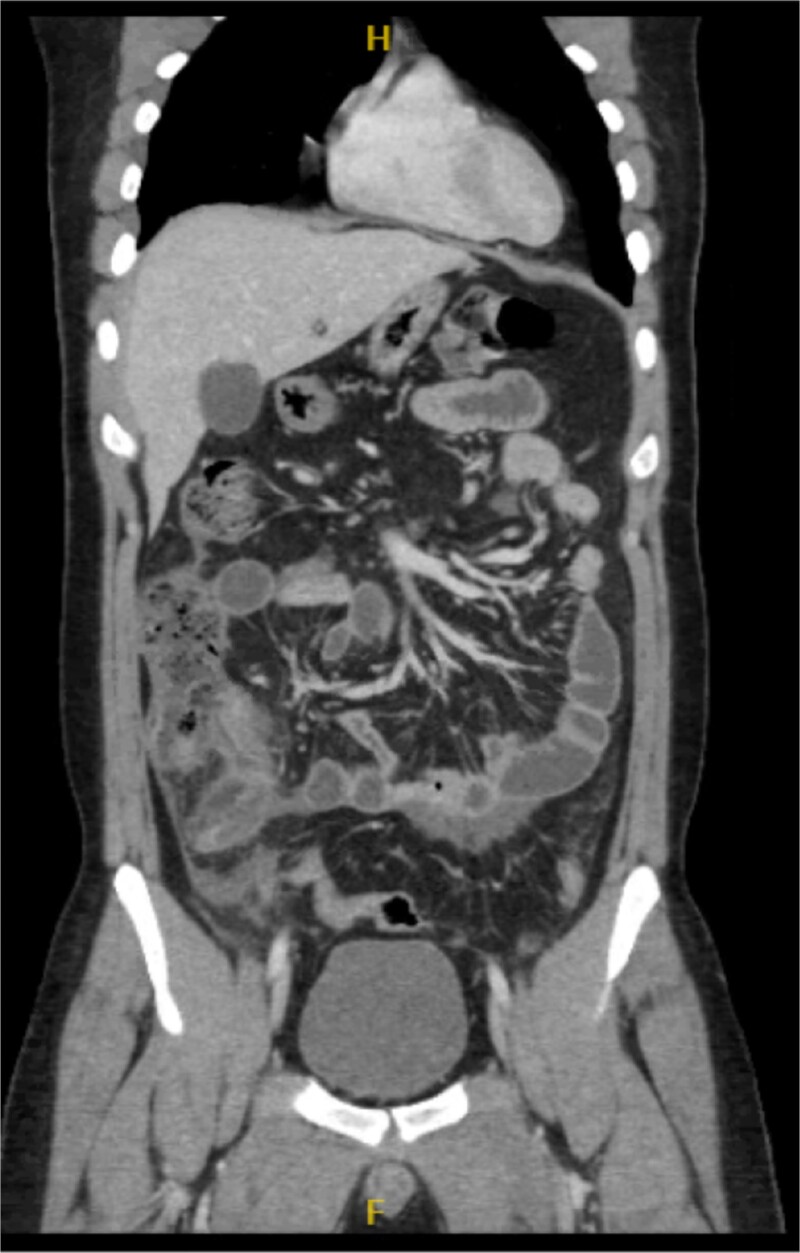
Coronal view of the inflamed appendix with phlegm formation.

**Figure 2:**
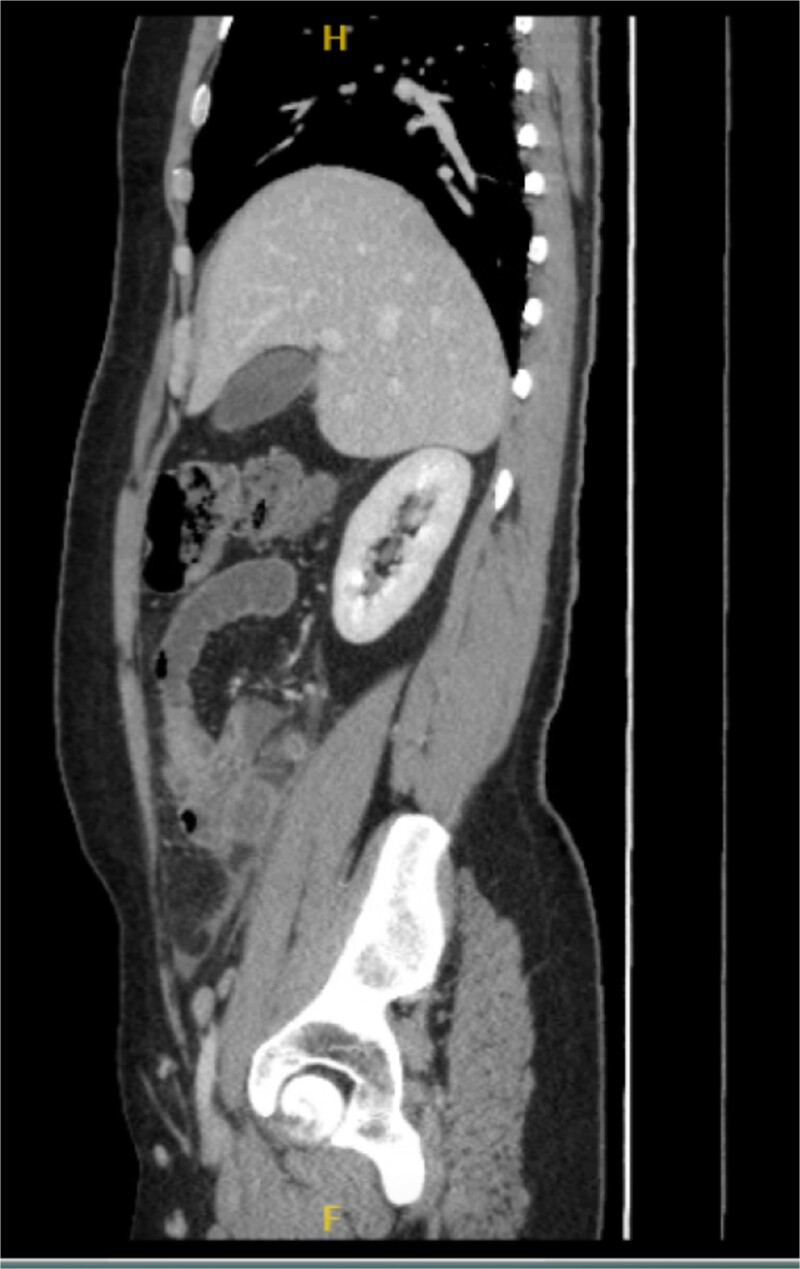
Sagittal view of the inflamed appendix with phlegm formation.

**Figure 3:**
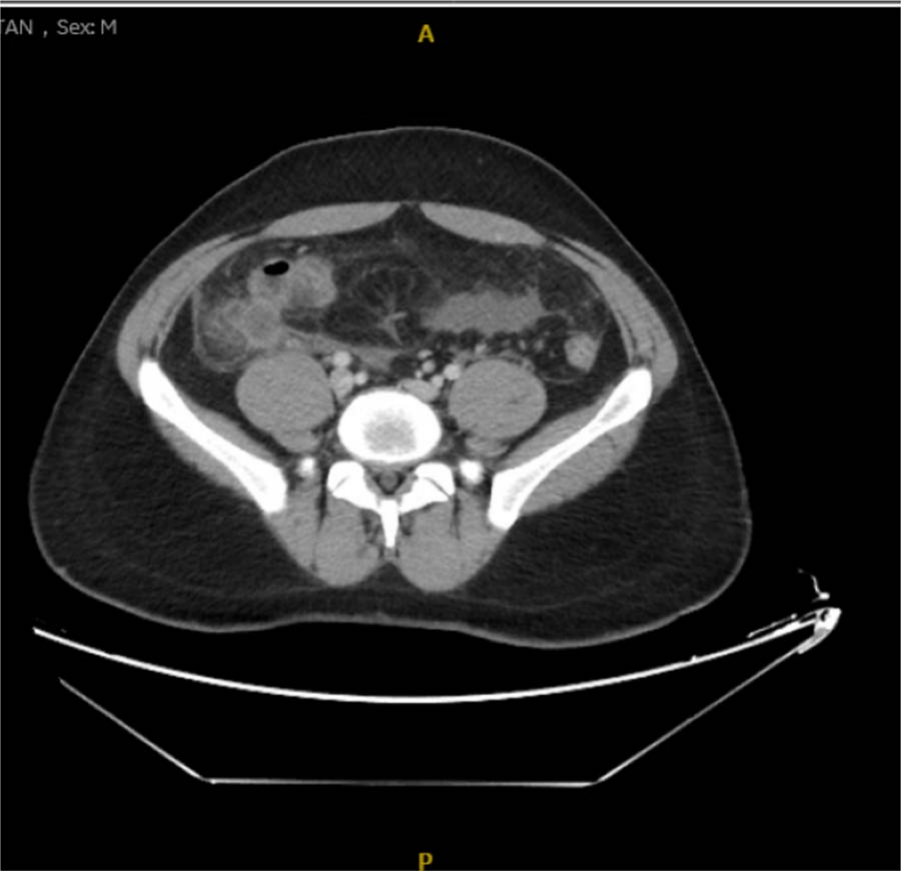
Axial view of the inflamed appendix with phlegm formation.

His laboratory tests revealed a white blood cell (WBC) count of 7.8 × 10^3^; all other blood workups were within normal ranges. An abdominal CT scan showed a dilated fluid field in the appendix measuring ~1.8 cm in diameter, with fat stranding, with few areas of decreased well enhancement, and a possible defect in the distal portion of the appendix with phlegm formation (Figs 1–3). All other organs, including the spleen, were unremarkable for any positive findings, with no evidence of free fluid or any splenic injury at the time of the CT scan. The patient was admitted under general surgical care, started on intravenous antibiotics, and was scheduled for laparoscopic exploration and appendectomy. Intraoperative findings included an inflamed appendix and perforation at the midpoint. The patient had an uneventful recovery. The patient was discharged in good condition on postoperative Day 2, with follow-up in the outpatient clinic.

Histopathological examination of the sample revealed acute perforated appendicitis and peri appendicitis.

## Discussion

Acute appendicitis is considered an emergency condition, occurring primarily due to luminal obstruction of the appendix caused by stool, mucus plugs, or foreign bodies [2]. Acute nonobstructive appendicitis can result from bacterial invasion of the lymphoid tissue of the appendicular wall [4]. The relationship between blunt abdominal trauma and acute appendicitis is debatable. This debate was first reported in the 1930s, and multiple reports have been published thereafter [5]. Despite early reporting, cases describing the association between blunt trauma and acute appendicitis are scarce. Intestinal injury resulting from blunt abdominal trauma has an incidence rate of 5%–15%, whereas appendicular injury is not thoroughly reported [1]. A systematic review reported 28 cases of traumatic appendicitis that occurred between 1991 and 2009 [6, 7]. Hennington and associates reported two cases of acute appendicitis induced and preceded by blunt trauma. In both cases, the possible mechanism of appendicitis was appendiceal obstruction from edema, inflammation, and lymphoid tissue hyperplasia [8]. The pathophysiology of traumatic appendicitis is unclear; however, it likely results from multiple mechanisms. These factors include direct compression or crush injury to the lumen of the appendix, shearing traumatic injuries, indirect obstruction of the appendiceal lumen caused by an ileocecal hematoma, or the traumatic impaction of stool within the appendix. Another theory suggests that blunt abdominal trauma results in edema of the appendiceal wall, along with inflammation and hyperplasia of the lymphoid tissue, ultimately leading to obstruction of the appendiceal lumen and acute appendicitis [8]. Overall, blunt abdominal trauma typically results in elevated intra-abdominal pressure, consequently causing an increase in intracecal pressure and appendix distension, possibly leading to appendicitis [5, 6–9]. Our research indicates that direct injury to the appendix often occurs alongside injuries to other intra-abdominal organs; however, the appendix is rarely injured in isolation from direct trauma due to its high mobility and small size [3]. Patients generally exhibit symptoms akin to nontraumatic appendicitis, including abdominal pain, nausea, loss of appetite, and fever. An abdominal examination typically reveals tenderness in the RLQ and signs of peritonitis. The diagnostic criteria for traumatic appendicitis include a previously asymptomatic patient and evidence of abdominal trauma, with the onset of persistent and progressive symptoms occurring between 6 and 48 h after trauma, ultimately confirmed as appendicitis through surgical intervention [10, 11].

Radiological findings in CT images revealed signs of appendicitis: an enlarged appendicular diameter >6 mm, an appendicular wall thickness >2 mm, mesenteric fat stranding, the presence of an appendicolith, and signs of complications, including perforation or the formation of a peri-appendiceal abscess [10–12]. Careful assessment requires careful medical history taking, physical examination, and radiological investigations with ultrasound or CT scans, which are more sensitive and specific. A high index of suspicion of post-traumatic appendicitis is required because of the rarity of the condition. Treatment includes intravenous antibiotic administration and appendectomy.

## Conclusions

Appendicitis may occur after sustaining abdominal trauma. For any suspected appendicitis after blunt abdominal injury, surgical exploration might be preferred to identify other injuries, including bowel injuries.
